# Is There a Correlation Between Left Ventricular Outflow Tract Velocity Time Integral and Stroke Volume Index in Patients Undergoing Cardiac Surgery?

**DOI:** 10.7759/cureus.27257

**Published:** 2022-07-25

**Authors:** Hatsuo Isogai, Osamu Ogasawara

**Affiliations:** 1 Emergency and Critical Care Medicine, Kariya Toyota General Hospital, Kariya, JPN

**Keywords:** lvot vti, stroke volume index, pulmonary artery catheter, transesophageal echocardiography (tee), velocity time integral, left ventricular outflow tract

## Abstract

Introduction

Left ventricular outflow tract velocity time integral (LVOT VTI) is a promising surrogate for stroke volume (SV). However, there is controversy in the literature regarding its correlation with thermodilution or newer cardiac output measurement techniques. This study was conducted to determine the correlation between LVOT VTI determined by transesophageal echocardiography (TEE) with stroke volume index (SVI) calculated by thermodilution.

Methods

Consecutive patients older than 17 years undergoing elective cardiac surgery with pulmonary artery catheter (PAC) and TEE monitoring between September 2021 and February 2022 were included in this prospective, descriptive, single-center study. LVOT VTI was measured using TEE after induction of anesthesia but before skin incision and at least four hours after initial LVOT VTI measurement. SVI was simultaneously measured using the continuous thermodilution technique with a PAC. The correlation between LVOT VTI and SVI was determined with Pearson’s correlation index.

Results

Twelve patients were included and 21 paired measurements were compared. Mean SVI was 31.62 ± 10.71 mL/m^2^ and mean LVOT VTI was 14.74 ± 4.79 cm. The Pearson's correlation index for the two measurements was r = 0.257, p = 0.262.

Conclusion

This prospective study demonstrated a weak correlation between LVOT VTI and SVI in patients undergoing cardiac surgery.

## Introduction

Stroke volume (SV) is a basic hemodynamic parameter. Thermodilution using a pulmonary artery catheter (PAC) is a reliable method for monitoring SV. However, the use of PACs does not lead to better outcomes [1]. PACs are invasive; therefore, the routine use of PACs is discouraged [2-4]. In contrast, echocardiography plays an important role in hemodynamic monitoring and is less invasive. The guidelines from the American Society of Echocardiography recommended that the left ventricular outflow tract (LVOT) be used to measure SV [5]. Calculation of SV using echocardiography requires the LVOT velocity time integral (VTI) and the LVOT cross-sectional area (CSA) measured. The CSA is calculated as LVOT anterior-posterior diameter (cm)^2^ x 0.785, based on the assumption of LVOT CSA being a circular orifice. However, conflicting evidence has been reported concerning the correlation between SV measured by PAC and echocardiography [6-8].

SV measured by echocardiography may be inaccurate due to measurement errors in LVOT CSA. Several factors contribute to the miscalculation of LVOT CSA. First, minor measurement errors in LVOT diameter due to poor acoustic window and technical problems are squared in the process of determining LVOT CSA [9]. Second, the calculation is based on the assumption that the LVOT is a circular orifice [10]. This assumption leads to a 15% underestimation of LVOT CSA because LVOT is an elliptical orifice, not a circular orifice.

Recently, LVOT VTI has been proposed as a surrogate to estimate stroke volume index (SVI) based on the assumption that the LVOT diameter is constant in each person [5]. However, a previous study demonstrated that LVOT VTI measured by transthoracic echocardiography (TTE) and SVI measured using a PAC or a pulse index contour cardiac output (PiCCO) monitor did not correlate well in intensive care unit patients [11]. Transesophageal echocardiography (TEE) is the more precise method to calculate cardiac output (CO) [6]. TEE provides more accurate images compared to TTE, and VTI measurement with TEE may be more accurate. The aim of this study was to evaluate the correlation between LVOT VTI measured by TEE and SVI measured using a PAC in patients undergoing cardiac surgery.

## Materials and methods

This prospective, descriptive study received ethics approval from the institutional investigational committee at the principal investigator’s hospital (Approval Number: 703). Informed consent was obtained from all patients or their relatives. The study was carried out in Kariya Toyota General Hospital, Kariya, Japan.

From September 2021 to February 2022, consecutive patients older than 17 years undergoing elective cardiac surgery with PAC and TEE monitoring were enrolled in the study. The exclusion criteria were: more than mild aortic regurgitation (AR); more than moderate tricuspid regurgitation (TR); subaortic obstruction; inability to advance TEE into the stomach; atrial fibrillation (AF); use of intra-aortic balloon pump; use of veno-arterial extracorporeal membrane oxygenation; and declined informed consent.

Anesthetic management was performed at the discretion of the attending anesthesiologist. After induction of general anesthesia and tracheal intubation, the TEE probe (Epiq 7; Koninklijke Philips N.V., Amsterdam, Netherlands) was inserted. The absence of significant AR, TR, and AF and a heart rate (HR) between 40 and 180 beats per minute were confirmed. Then, LVOT VTI was measured by a single anesthesiologist, who was blinded to the PAC results.

Measurements of LVOT VTI were performed after induction of anesthesia but before skin incision and at least four hours after initial LVOT VTI measurement. LVOT VTI was not measured during cardiopulmonary bypass. Patients were included in the study when the exclusion criteria were no longer present due to surgery (e.g., a patient with severe AR was included after aortic valve repair when the original disease was treated.). LVOT VTI was recorded from the TEE deep transgastric long-axis view or the transgastric basal long-axis view if the Doppler beam flow angle was less than 20 degrees. The sample volume was positioned 5-10 mm away from the aortic valve annulus. The outer edge traces of LVOT VTI were measured. Three consecutive samples were gathered and the results were averaged. Measurements were conducted under stable hemodynamic and cardiac rhythm conditions. If HR changed more than 20%, then measurements were terminated and only resumed after HR was stable.

SVI was determined using a PAC for continuous pulmonary artery thermodilution. The PAC was equipped with a thermal filament to heat the blood in the right ventricle and changes in blood temperature were detected. SVI was continuously calculated, based on blood temperature changes. SVI was measured after completing the LVOT VTI measurements. The measurement of LVOT VTI took about one minute. SVI measurements using a PAC averaged about one to three minutes; thus, the measurement time gap was approximately one to two minutes. In addition to LVOT VTI and SVI measurements, we recorded sex, age, height, body weight, base ejection fraction, and the type of surgery.

Statistical analysis

Quantitative variables are expressed as mean ± standard deviation or median and interquartile range. The Pearson correlation index, concordance, and the intraclass correlation coefficient (ICC) were used to assess the correlation between SVI and LVOT VTI. All statistical analyses were performed with EZR (Easy R) (Saitama Medical Center, Jichi Medical University, Saitama, Japan), which is a modified version of R commander [12].

## Results

From September 1, 2021 to February 25, 2022, 18 patients were screened for inclusion in the study. Six patients were excluded from the study, leaving 12 patients. Reasons for exclusion included the inability to place the PAC (2) and more than moderate AR (2) or AF (2). Finally, 21 paired measurements were compared.

**Figure 1:**
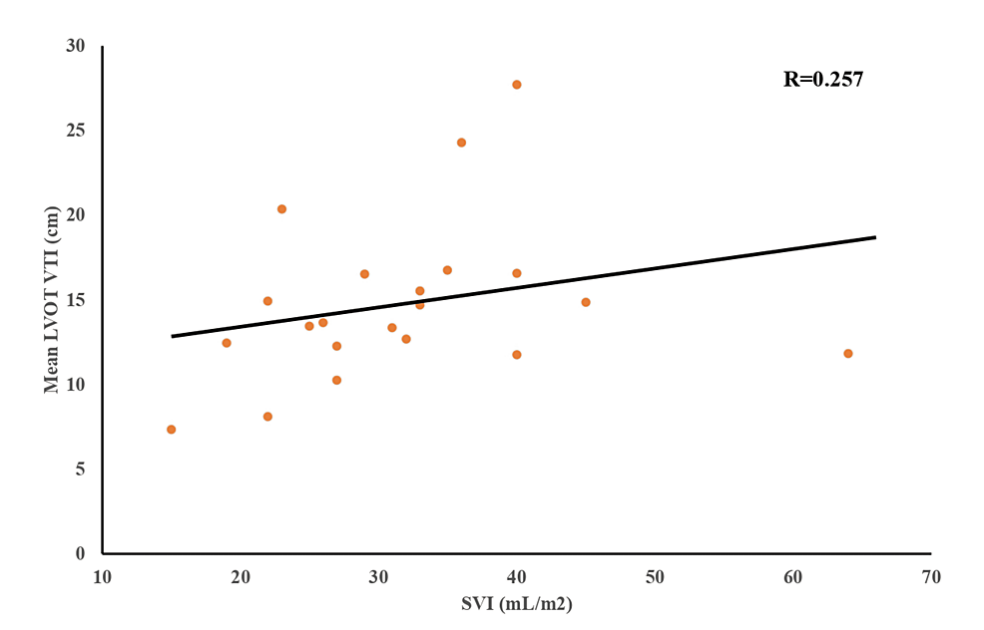
Correlation between mean LVOT VTI and SVI LVOT VTI: left ventricular outflow tract velocity time integral; SVI: stroke volume index

Table 1 shows the baseline characteristics of the 12 patients. A summary of surgical procedures is presented in Table 2. Mean SVI was 31.62 ± 10.71 mL/m^2^ and mean LVOT VTI was 14.74 ± 4.79 cm. The Pearson correlation index for these variables was r = 0.257 (p = 0.262) (Figure 1). The ICC for the two measurements of VTI was 0.96.

**Table 1:** Baseline characteristics IQR: interquartile range; BMI: body mass index; EF: ejection fraction

Characteristics	Overall (N=12)
Age (years), median (IQR)	68 (58-73)
Gender, male (%)	7 (58)
Height (cm), median (IQR)	165 (157-170)
Weight (kg), median (IQR)	59.9 (49.2-80.7)
BMI (kg/m^2^), median (IQR)	21.9 (19.2-26.6)
EF (%), median (IQR)	54 (43-67)

**Table 2:** Surgical intervention

Intervention	Overall (N=12)
Aortic valve repair	3
Mitral valve repair	1
Dual valve repair	1
Coronary artery bypass grafting	4
Total arch replacement	2
Ascending aortic replacement	1

## Discussion

In this prospective trial, we demonstrated a weak correlation between LVOT VTI determined via TEE and SVI measured using a PAC. The study patients underwent general cardiac surgery, and the type of surgery varied. Intra-rater reliability was high, based on the ICC.

To the best of our knowledge, this is the first study comparing LVOT VTI measured via TEE with SVI measured by continuous thermodilution. A previous study demonstrated a lack of correlation between LVOT VTI measured by TTE and SVI measured by PAC or PiCCO [11]. We hypothesized that the limited view using TTE contributed to the poor correlation between LVOT VTI and SVI. TEE gives better views compared with TTE. Thus, we speculated that LVOT VTI measured using TEE would correlate better with SVI. Several studies have demonstrated the usefulness of cardiac parameters estimated by echocardiography, especially TEE. A meta-analysis demonstrated the superior accuracy of TEE for estimating CO compared to TTE [6]. However, we did not show a strong correlation between LVOT VTI and SVI, even when LVOT VTI was measured using TEE.

There are several factors affecting our results. The subjects in our study were undergoing cardiac surgery. Several studies have demonstrated the usefulness of LVOT VTI as a surrogate marker for CO because LVOT CSA correlates with physical characteristics, such as height and body surface area [13]. However, these studies were conducted on healthy individuals and the results may not apply to patients with cardiac diseases. The structure and motion of the heart in patients with cardiac disease are not normal [14]. Surgical intervention around the mitral or aortic valve might also affect the size of the LVOT CSA. A recent study showed that mitral valve surgery reduces the LVOT CSA, and the smaller the size of the implanted device, the more reduction in LVOT CSA [15]. Therefore, LVOT CSA should be considered in patients undergoing cardiac surgery when calculating SVI, and LVOT VTI alone may not estimate SVI. The planimetry of CSA may be better for calculating CSA than using an LVOT diameter in the mid-esophageal long-axis view because LVOT is not circular but elliptical [16]. Although the 3D image is a promising option, 2D planimetry correlated better with the PAC than 3D images [17].

Anatomical heterogeneity in patients undergoing cardiac surgeries may have influenced our study because maintaining the echo beam flow angle close to 0 degrees was relatively difficult. In TEE, the deep transgastric long-axis view or the transgastric basal long-axis view is recommended for obtaining the appropriate LVOT. In some cases, we managed to obtain a proper view to measure the LVOT VTI, even with the view described above. Some of the views may have been insufficient. Therefore, we cannot rule out the chances of underestimating LVOT VTI because of technical problems. However, we occasionally encounter a population in which we cannot obtain the proper view. Therefore, we thought by including such a population, we could adapt our study to more realistic clinical situations.

We adopted continuous pulmonary artery thermodilution based on the data in our daily clinical practice. A recent study suggested that the continuous method barely passed the interchangeability criteria with the intermittent method [18]. In addition, the continuous method was more accurate and less variable compared with the intermittent method [19].

Most studies used intermittent measurements, probably to perform the thermodilution and echocardiography simultaneously. Continuous pulmonary artery thermodilution averages SVI over one to three minutes. However, when hemodynamic instability occurs, more time is needed to calculate SVI (usually more than six minutes). Although we conducted SVI measurements under hemodynamically stable conditions, we did not determine the length of time to calculate SVI, which determines the time gap between each method. Therefore, the three LVOT VTI measurements may not show the average SVI during PAC measurements.

There was a limitation to this study. This study might be underpowered to detect outcomes due to the small sample size. The expected sample size could not be obtained due to coronavirus disease 2019 (COVID-19). Considering the low correlation, we estimated that showing a strong correlation would be difficult, even if more patients were included. 

Based on the results of this study, LVOT VTI measured by TEE cannot replace SVI. Thus, estimating accurate CO with echocardiography might be difficult at this point. However, as the LVOT CSA is constant in each person, any change in the SV might be the result of a change in LVOT VTI. Therefore, tracking the rate of change in LVOT VTI allows evaluation of response to fluid challenge, vasopressor therapy, and inotropic therapy [9]. In this respect, LVOT VTI may be useful.

## Conclusions

Our study demonstrated a weak correlation between LVOT VTI measured by TEE and SVI calculated using a PAC in a small sample of patients undergoing cardiac surgery. While serial measurements of LVOT VTI are useful in practice for assessing the changes in SV with treatments, one point measurement of LVOT VTI is unlikely to accurately correlate with SVI.

## References

[1] Sandham JD, Hull RD, Brant RF (2003). A randomized, controlled trial of the use of pulmonary-artery catheters in high-risk surgical patients. N Engl J Med.

[2] Hadian M, Pinsky MR (2006). Evidence-based review of the use of the pulmonary artery catheter: impact data and complications. Crit Care.

[3] Kearney TJ, Shabot MM (1995). Pulmonary artery rupture associated with the Swan-Ganz catheter. Chest.

[4] Ikuta K, Wang Y, Robinson A, Ahmad T, Krumholz HM, Desai NR (2017). National trends in use and outcomes of pulmonary artery catheters among medicare beneficiaries, 1999-2013. JAMA Cardiol.

[5] Porter TR, Shillcutt SK, Adams MS, Desjardins G, Glas KE, Olson JJ, Troughton RW (2015). Guidelines for the use of echocardiography as a monitor for therapeutic intervention in adults: a report from the American Society of Echocardiography. J Am Soc Echocardiogr.

[6] Zhang Y, Wang Y, Shi J, Hua Z, Xu J (2019). Cardiac output measurements via echocardiography versus thermodilution: A systematic review and meta-analysis. PLoS One.

[7] Mercado P, Maizel J, Beyls C (2017). Transthoracic echocardiography: an accurate and precise method for estimating cardiac output in the critically ill patient. Crit Care.

[8] Wetterslev M, Møller-Sørensen H, Johansen RR, Perner A (2016). Systematic review of cardiac output measurements by echocardiography vs. thermodilution: the techniques are not interchangeable. Intensive Care Med.

[9] Blanco P (2020). Rationale for using the velocity-time integral and the minute distance for assessing the stroke volume and cardiac output in point-of-care settings. Ultrasound J.

[10] Doddamani S, Bello R, Friedman MA (2007). Demonstration of left ventricular outflow tract eccentricity by real time 3D echocardiography: implications for the determination of aortic valve area. Echocardiography.

[11] Blancas R, Martínez-González Ó, Ballesteros D (2019). Lack of correlation between left ventricular outflow tract velocity time integral and stroke volume index in mechanically ventilated patients. Med Intensiva (Engl Ed).

[12] Kanda Y (2013). Investigation of the freely available easy-to-use software 'EZR' for medical statistics. Bone Marrow Transplant.

[13] Goldman JH, Schiller NB, Lim DC, Redberg RF, Foster E (2001). Usefulness of stroke distance by echocardiography as a surrogate marker of cardiac output that is independent of gender and size in a normal population. Am J Cardiol.

[14] Mehrotra P, Jansen K, Flynn AW, Tan TC, Elmariah S, Picard MH, Hung J (2013). Differential left ventricular remodelling and longitudinal function distinguishes low flow from normal-flow preserved ejection fraction low-gradient severe aortic stenosis. Eur Heart J.

[15] Rosendal C, Hien MD, Bruckner T, Martin EO, Szabo G, Rauch H (2012). Left ventricular outflow tract: intraoperative measurement and changes caused by mitral valve surgery. J Am Soc Echocardiogr.

[16] Montealegre-Gallegos M, Mahmood F, Owais K, Hess P, Jainandunsing JS, Matyal R (2014). Cardiac output calculation and three-dimensional echocardiography. J Cardiothorac Vasc Anesth.

[17] Canty DJ, Kim M, Guha R (2020). Comparison of cardiac output of both 2-dimensional and 3-dimensional transesophageal echocardiography with transpulmonary thermodilution during cardiac surgery. J Cardiothorac Vasc Anesth.

[18] Kouz K, Michard F, Bergholz A (2021). Agreement between continuous and intermittent pulmonary artery thermodilution for cardiac output measurement in perioperative and intensive care medicine: a systematic review and meta-analysis. Crit Care.

[19] Bootsma IT, Boerma EC, Scheeren TW, de Lange F (2022). The contemporary pulmonary artery catheter. Part 2: measurements, limitations, and clinical applications. J Clin Monit Comput.

